# A Novel *Leptospira interrogans* Protein LIC13086 Inhibits Fibrin Clot Formation and Interacts With Host Components

**DOI:** 10.3389/fcimb.2021.708739

**Published:** 2021-07-01

**Authors:** Felipe José Passalia, Marcos Bryan Heinemann, Mônica Larucci Vieira, Ana Lucia T. O. Nascimento

**Affiliations:** ^1^ Laboratório de Desenvolvimento de Vacinas, Instituto Butantan, São Paulo, Brazil; ^2^ Programa de Pós-Graduação Interunidades em Biotecnologia, Instituto de Ciências Biomédicas, Universidade de São Paulo, São Paulo, Brazil; ^3^ Laboratório de Zoonoses Bacterianas, Faculdade de Medicina Veterinária e Zootecnia, Universidade de São Paulo, São Paulo, Brazil; ^4^ Departamento de Microbiologia, Instituto de Ciências Biológicas (ICB), Universidade Federal de Minas Gerais (UFMG), Belo Horizonte, Brazil

**Keywords:** *Leptospira*, leptospirosis, recombinant protein, host pathogen interaction, fibrin clot inhibition

## Abstract

Leptospirosis is a neglected zoonosis, caused by pathogenic spirochetes bacteria of the genus *Leptospira*. The molecular mechanisms of leptospirosis infection are complex, and it is becoming clear that leptospires express several functionally redundant proteins to invade, disseminate, and escape the host’s immune response. Here, we describe a novel leptospiral protein encoded by the gene LIC13086 as an outer membrane protein. The recombinant protein LIC13086 can interact with the extracellular matrix component laminin and bind plasminogen, thus possibly participating during the adhesion process and dissemination. Also, by interacting with fibrinogen and plasma fibronectin, the protein LIC13086 probably has an inhibitory effect in the fibrin clot formation during the infection process. The newly characterized protein can also bind molecules of the complement system and the regulator C4BP and, thus, might have a role in the evasion mechanism of *Leptospira*. Taken together, our results suggest that the protein LIC13086 may have a multifunctional role in leptospiral pathogenesis, participating in host invasion, dissemination, and immune evasion processes.

## Introduction

Human leptospirosis is an emerging neglected disease and occurs mainly in tropical and subtropical regions where the transmission conditions are appropriate. It’s estimated more than one million cases and approximately 60 thousand deaths worldwide every year (Costa et al., 2015). Leptospirosis is caused by pathogenic Gram-negative bacteria of the genus *Leptospira*, which are classified in more than 90 genetic species and divided serologically into more than 300 serovars (Vincent et al., 2019). The synanthropic rodents *Rattus rattus* and *Rattus norvegicus* are the main urban reservoirs of the bacteria, being chronic asymptomatic carriers. Leptospires colonize their kidneys and are excreted alive in the environment. Humans are infected mainly *via* cuts and abrasions on the skin when exposed to contaminated soil or water (Ko et al., 2009). Early symptoms of leptospirosis are unspecific and include fever, headaches, mild and muscle aches, and are commonly misdiagnosed by other febrile diseases. In some cases, leptospirosis progresses to severe conditions characterized by jaundice, hemorrhages, hypotension, and multiple organ failure. These include leptospirosis-associated severe pulmonary hemorrhage syndrome (Trevejo et al., 1998) and Weil’s disease (Levett, 2001).

The initial process of infection consists of leptospires’ adhesion onto extracellular matrix molecules in the host epithelial skin tissue. This step is mediated by leptospiral outer membrane adhesins, of which several were already characterized (Vieira et al., 2014; Figueredo et al., 2017; Pereira et al., 2017; Cavenague et al., 2019; Kochi et al., 2019; Rossini et al., 2019; Passalia et al., 2020a). When leptospires reach the bloodstream, they are able to interact with circulating host plasma components (Vieira et al., 2020). Leptospires bind to the zymogen plasminogen, which is converted into plasmin (Vieira et al., 2012). This bacterial surface-associated plasmin functions as a mechanism of degradation of several host components, immune evasion, and dissemination (Vieira et al., 2009; Vieira et al., 2011; Vieira et al., 2012; Vieira et al., 2013; Vieira and Nascimento, 2016). Surface exposed protein in leptospires can interact with human fibrinogen leading to a partial inhibition of fibrin clot formation thus facilitating the dissemination (Oliveira et al., 2013; Fernandes et al., 2015). Furthermore, several leptospiral surface proteins interact with regulators and components of the host complement system as a mechanism of innate immune evasion (Siqueira et al., 2017; Cavenague et al., 2019; Kochi et al., 2019; Passalia et al., 2020a).

In the present work, we characterize a novel leptospiral outer membrane protein encoded by the gene LIC13086, and evaluate its ability to bind human extracellular matrix and plasma components. The *in vitro* assays with the recombinant protein point to a probable multifunctional role of LIC13086 during the infection process of leptospirosis.

## Material and Methods

### Ethics Statement

Animal experimentation adopts the guidelines of the Brazilian College of Animal Experimentation (COBEA) and was approved by the Butantan Institute’s Ethics Committee on Animal Use (São Paulo; protocol CEUAIB 3431090117).

### Bacterial Strain

The virulent pathogenic bacteria *L. interrogans* serovar Copenhageni strain Fiocruz L1-130 and culture-attenuated *L. interrogans* serovar Copenhageni strain M20 were cultured at 30°C under aerobic conditions in liquid EMJH medium (Difco) supplemented with 10% Leptospira Enrichment EMJH (Difco). Virulence of *L. interrogans* serovar Copenhageni (strain Fiocruz L1-130) was maintained by sequential passages in Golden Syrian hamsters (Faine et al., 1999). *E. coli* DH5a and *E. coli* BL21 (DE3) Star pLysS (Invitrogen) were used for cloning and recombinant protein expression, respectively.

### Leptospirosis Patients’ Serum Samples

Patients were diagnosed with leptospirosis by the microscopic agglutination test (MAT) with four-fold titers increase between samples from 10 to 15 days interval. The serum samples correspond to onset (MAT-negative; MAT-) or convalescent phase (MAT-positive; MAT+) of the disease. The cutoff value was determined by the mean absorbance plus three times the standard deviation of the values obtained with the sera from healthy donors, as previously described (Vieira et al., 2010b; Fernandes et al., 2012; Bourhy et al., 2013; Vedhagiri et al., 2013).

Serum samples from the convalescent phase (MAT+) were obtained from the serum collection of Instituto Adolfo Lutz, São Paulo, Brazil. The serum samples were donated for research purposes.

### Biological Components

Laminin (L2020), cellular fibronectin (F2518), collagen type I (C3867), collagen type IV (C0543), elastin (E6902), e-cadherin (5085), condhroitin (C9819), heparan sulfate (H7640), heparin (SRE0027), condhroitin-4 (27042), plasminogen (P7999), plasma fibronectin (F2006), vitronectin (V8379), fibrinogen (F4883), thrombin (T6884), fetuin (F3004), and BSA (A3912) were obtained from Sigma-Aldrich. Factor H (341274) purified from human serum was purchased from EMD Chemicals. C3b (A114), C4b (A108), C4BP (A109), C5b6 (A122), C6 (A123), C7 (A124), C8 (A125), and C9 (A126) isolated from normal human serum were acquired from Complement Technology.

### *In Silico* Analysis of LIC13086

The LIC13086 (LIC_RS15890) coding sequence was selected from the genome sequence of *L. interrogans* serovar Copenhageni (Nascimento et al., 2004a) based on the cellular location prediction by CELLO, http://cello.life.nctu.edu.tw (Yu et al., 2006). The presence of signal peptide was evaluated by LipoP, http://www.cbs.dtu.dk/services/LipoP/ (Juncker et al., 2003), the predicted structural and functional domains of the protein were analyzed by SMART, http://smart.embl-heidelberg.de (Letunic et al., 2015), and TOPCONS, https://topcons.cbr.su.se/pred/ (Tsirigos et al., 2015). Multiple sequence alignment was performed by Clustal Omega (Madeira et al., 2019) and the tertiary model of the protein was predicted by I-TASSER server (Zhang, 2008).

### RNA Extraction and Real-Time PCR

Cultures of virulent or culture-attenuated *L. interrogans* serovar Copenhageni with a concentration of 5 × 10^8^ cell/ml were centrifuged at 2,000 × *g*. The pellet was resuspended in 1 ml of TRIzol^™^ and 260 μl of chloroform was added. The aqueous phase was separated after centrifugation (12,000 × *g*), followed by the addition of isopropanol (660 μl). The samples were centrifuged (12,000 × *g*), the total RNA was precipitated by the addition of ethanol (75%) and resuspended in RNAse-free water. The remaining contaminating DNA was eliminated by incubation with 2 μl of DNAseI (Invitrogen). Finally, the synthesis of the complementary DNA (cDNA) was performed using 1 μg of purified RNA as template with SuperScript™ III Reverse Transcriptase kit (ThermoFisher), according to the manufacturer’s instructions.

Real-time PCR was performed in a C1000 Touch™ Thermal Cycler (Bio-Rad) using Power SYBR^®^ Green PCR Master Mix (Applied Biosystem). The cDNAs were used for quantification of LIC1306 and 16S transcripts using specific pairs of primers (**Table 1**). The conditions for amplification were 95°C for 10 min; 40 cycles at 95°C for 15 s followed by 60°C for 60 s. The ribosomal 16S was used as the control expression.

**Table 1:** Sequence of primers used in cloning and qPCR.

Gene	Function	Oligonucleotides
LIC13086	Cloning	Forward 5’ CTCGAGTGTACAAATTCTCAAGATCCCAAC
		Reverse 5’ AAGCTTTCAGTTCGATGCAGGAAATACTTC
LIC13086	qPCR	Forward 5’ CAAGGGAAGTTTTACAATCG
		Reverse 5’ CCGTATCTGGAAGAATTACG
16S	qPCR	Forward 5’GGTGCAAGCGTTGTTCGG
		Reverse 5’ GATATCTACGCATTTCACCGC

### Cloning, Expression, and Purification of LIC13086

The gene was amplified without the signal peptide by PCR, using the genomic DNA of *L. interrogans* serovar Copenhageni L1-130 as template using the pairs of primers described in **Table 1**. The amplified PCR product was cloned into PGEM-T-Easy vector (Promega) and subsequently cloned into pAE expression vector (Ramos et al., 2004) at the restriction sites *XhoI* and *HindIII*. This plasmid contains encodes for six histidine residues at the N-terminal region of the recombinant protein, enabling the metal affinity chromatography purification. The cloned insert was verified by automated sequencing. Chemically competent *E. coli* BL21 (DE3) Star pLysS cells were transformed with pAE-LIC13086 plasmid. *E. coli* cells were grown at 37°C in Luria-Bertani (LB) liquid medium and the protein expression was induced by the addition of 0.5 mM IPTG (Isopropyl β-D-1-thiogalactopyranoside). The recombinant protein was expressed in the insoluble fraction, which was solubilized in denaturation buffer (50 mM Tris-HCl pH 7.4; 200 mM NaCl; 8 M urea) and refolded by slow dilution in the same buffer without urea. The purification was performed by immobilized metal affinity chromatography (IMAC) in nickel charged Chelating Sepharose column (GE Healthcare). The eluted recombinant protein was buffer-exchanged to PBS by dialysis and evaluated by SDS-PAGE (12%).

### Circular Dichroism Spectroscopy

The recombinant protein was dialyzed against 10 mM sodium phosphate buffer (pH 7.4) and the spectroscopy measurement was performed in Jasco J-810 spectropolarimeter (Japan Spectroscopic) equipped with a Peltier for temperature control, with a 1 mm path length cell. Measured spectra represent the average of 10 scans recorded from 180 to 260 nm in a 0.1 nm interval at 25°C. Additionally, the purified recombinant protein was treated with 3 mM EDTA for 15 min at room temperature and dialyzed against 10 mM sodium phosphate buffer (pH 7.4). The protein was maintained in the same buffer (Lee et al., 2018; Raith et al., 2019). After dialysis, 0.5 mM CaCl_2_, 1 mM CaCl_2_, or 1 mM MgCl_2_ were added to the recombinant protein, followed by circular dichroism analysis. The spectra are expressed in residual molar ellipticity. Spectra data was submitted to BeStSel web server for secondary structure content analysis (Micsonai et al., 2018).

### Recombinant Protein Reactivity With Serum Samples of Patients Diagnosed With Leptospirosis

The recombinant protein LIC13086 was immobilized onto ELISA plates (High Binding, Sarstedt) at 250 ng/well or 1 μg/well for 16 h at 4°C. The wells were washed three times with PBS containing 0.05% Tween-20 (v/v) (PBS-T) and blocked with PBS-T with 10% skimmed dry milk (w/v) (PBS-T-Milk) for 2 h at 37°C. Serum samples of leptospirosis patients at the convalescent phase (MAT+, n = 150) were diluted (1:100) in PBS-T-Milk and incubated for 1 h or 2 h at 37°C with the immobilized recombinant protein. After washing, the detection was performed by the incubation of HRP-conjugated anti-human IgG antibody (1:5,000) for 1 h at 37°C. Wells were washed and 1 mg/ml of o-phenylenediamine (OPD) in citrate-phosphate buffer (150 mM, pH 5.0) with 1 μl of H_2_O_2_ (30%). Reaction was incubated for 15 min at room temperature and stopped by the addition of 50 μl/well of 2 M H_2_SO_4_. The absorbance was determined in a microplate reader (Multiskan-FC, Thermo Fisher, Scientific, Helsinki, Finland) at 492 nm. Commercial normal human serum (NHS, Sigma-Aldrich) was used as control and the cut-off value was calculated based on the mean absorbance obtained with control serum plus three times the standard deviation (SD) between the same samples. Values above the cut-off were considered positive.

### Antiserum Production

Four to 6-week-old BALB/c female mice were immunized subcutaneously with 10 μg of recombinant protein mixed with 10% (v/v) Alhydrogel [2% Al(OH)3; Brenntag Biosector] and 10 μg of monophosphoryl lipid A (MPLA) as adjuvants. Two subsequent boost immunizations were given at 2-week intervals. Negative control mice were immunized with PBS and the adjuvants mix only. Animals were bled *via* submandibular vein and the pooled sera was evaluated by enzyme-linked immunosorbent assay (ELISA) for determination of antibody titers. Anti-recombinant protein sera were adsorbed with a suspension of *E. coli* to suppress the anti-*E. coli* antibodies reactivity (Gruber and Zingales, 1995). A western blot was performed with anti-LIC13086 serum (1:5,000) to confirm the detection of recombinant protein LIC13086.

### Assessment of Protein Location in *L. interrogans* by ELISA

Cultures of *L. interrogans* serovar Copenhageni strain M20 were centrifuged (2,000 × *g* for 15 min) and resuspended in PBS supplemented with 5 mM MgCl_2_. Approximately 6 × 10^8^ cell/well of either lysed or intact cells were coated onto ELISA plates overnight at 4°C. Plates were blocked with PBS-T-BSA (5% BSA) and incubated for 1 h at 37°C with anti-LIC13086 antiserum (1:100). After washing, plates were incubated with secondary HRP-conjugated goat anti-mouse IgG (1:5,000) for 1 h at 37°C. For the detection of the reactivity was performed by the addition of OPD as described before. The outer membrane protein LipL46 antibody (1:500) was used as positive control, and LipL31 (1:500) and DnaK (1:1,000) antibodies were used as negative controls.

### Flow Cytometry

*L. interrogans* serovar Copenhageni strain M20 cultures were centrifuged (2,000 × *g* for 15 min) and resuspended in PBS supplemented with 5 mM MgCl_2_. Cells were fixed with 2% paraformaldehyde for 1 h at 30°C. The leptospires were incubated with anti-LIC13086 antiserum (1:100) for 1 h at 30°C and, after washing, secondary FITC-conjugated goat anti-mouse IgG (1:50) was added for 2 h at 30°C. The measurements of fluorescence were performed in a BD FACSCanto II and data are expressed as median fluorescence intensity (MFI).

### Immunofluorescence

Cultures of *L. interrogans* serovar Copenhageni strain M20 were recovered by centrifugation at 2,000 × *g* for 15 min in PBS supplemented with 5 mM MgCl_2_. The leptospires were fixed with 2% paraformaldehyde, washed and blocked with PBS-T-BSA for 1 h at 30°C. The cells were incubated with anti-LIC13086 antiserum (1:100) for 1 h at 30°C. After washing, FITC-conjugated goat anti-mouse IgG (1:50) and DAPI (4′,6-Diamidine-2′-phenylindole dihydrochloride; Sigma-Aldrich) were added and incubated for 2 h at 30°C. Slides were prepared with ProLong Gold Antifade (Invitrogen, Carlsbad, California, USA) and the microscope images were captured in confocal immunofluorescence microscope LSM 510 META (Carl Zeiss).

### Binding of rLIC13086 to Host ECM, Plasma, and Complement System Components

Each component from ECM, plasma, or complement system was immobilized onto ELISA plates (1 μg/well) for 16 h at 4°C. For thrombin and vitronectin, 1 U or 250 ng were used, respectively. The negative controls BSA and fetuin were included. Plates were washed with PBS-T and blocked with PBS-T with 1% BSA (w/v) (PBS-T-BSA) for 1 h at 37°C. Then, 1 μg/well of recombinant protein was added, followed by incubation for 2 h at 37°C. After washing, recombinant protein was detected by incubation with mice polyclonal antisera (1:5,000) followed by HRP-conjugated anti-mouse IgG (1:5,000) or HRP-conjugated anti-His monoclonal antibody (1:10,000). The mean absorbance of each component was compared with the negative controls by Student’s t-test. A p-value below 0.05 was considered statistically significant.

For the determination of the dose-response curves, after coating of 1 μg/well of each component and blocking, different concentrations of rLIC13086 (0–5 μM/well) were added and incubated for 2 h at 37°C. The binding was detected with HRP-conjugated anti-His mAbs (1:20,000). The data were evaluated in GraphPad Prism software and the dissociation constant (K_D_) was calculated by fitting a non-linear regression curve of the specific binding equation to data.

### Assessment of Plasma and Complement System Components Recruitment by Recombinant Protein of Normal Human Serum

The recombinant protein was coated onto ELISA plates (1 μg/well) for 16 h at 4°C. BSA was used as a negative control. After 1 h blocking with PBS-T-BSA, the plates were incubated with solutions containing 3.75, 7.5, 15, and 30% commercial normal human serum (NHS) (Sigma-Aldrich) in PBS-T-BSA for 2 h at 37°C. After washing, human molecules binding to the immobilized recombinant protein was detected with antibodies against plasma fibronectin, plasminogen, fibrinogen, C7, C8, and C9 (at 1:10,000 dilution), followed by incubation with HRP-conjugated secondary antibodies.

### Recombinant Protein Binding Inhibition to Laminin by Sodium Metaperiodate Treatment

Additional characterization of sodium metaperiodate effect in laminin binding to LIC13086 was performed as previously described (Barbosa et al., 2006).

### Recombinant Protein Binding Inhibition to PLG by Aminocaproic Acid Co-incubation

Plasminogen (1 μg/well) was immobilized onto ELISA plates for 16 h at 4°C. After washing and blocking with PBS-T-BSA for 1 h at 37°C, recombinant protein LIC13086 was co-incubated with different concentrations of 6-aminocaproic acid (Sigma-Aldrich) (2 and 20 mM) for 1 h at 37°C. Detection was performed with HRP-conjugated anti-His mAb (1:10,000).

### Characterization of PLG Binding to Recombinant Protein and PLA Conversion

Recombinant protein LIC13086 (1 μg/well) was immobilized onto ELISA plates for 16 h at 4°C. Recombinant protein-associated plasmin generation was performed as previously described (Vieira et al., 2010a).

### Inhibition of Fibrin Clot Formation by Recombinant Protein

Recombinant protein LIC13086 (0.5 μg/μl in 200 mM NaCl) was mixed with fibrinogen (1 μg/μl in 0.85% NaCl) and incubated for 2 h at 37°C. After incubation, thrombin was added to a final concentration of 1 U per well and the fibrin clot formation was measured for 30 min with 1 min intervals in the absorbance of 595 nm.

### Characterization of rLIC13086 Binding to Complement Components

For evaluation of heparin interference in the rLIC13086 interaction with complement components, C7, C8, and C9 were immobilized onto ELISA plates (1 μg/well) for 16 h at 4°C. The wells were washed and blocked with PBS-T-BSA, different concentrations of heparin were co-incubated with 1 μg/well of rLIC13086 for 2 h at 37°C. After co-incubation, wells were washed and anti-HisTag mAb was incubated for 1 h at 37°C, followed by the detection by absorbance measure.

## Results

### Bioinformatics Analysis and Gene Expression of LIC13086

The gene coding for the protein LIC13086 was annotated as a putative lipoprotein in the genome of *L. interrogans* serovar Copenhageni L1-130 (Nascimento et al., 2004b). The protein LIC13086 was predicted to localize in the bacterial outer membrane by Cello software analysis, http://cello.life.nctu.edu.tw (Yu et al., 2006). According to LipoP software, http://www.cbs.dtu.dk/services/LipoP/ (Juncker et al., 2003), the protein contains an N-terminal region that can be recognized by the enzyme signal peptidase II (SpII), suggesting the membrane exportation and lipidation of the protein. When the LIC13086 sequence was analyzed by the SMART software, http://smart.embl-heidelberg.de (Letunic et al., 2015), a domain of unknown function (DUF4842) was identified. TOPCONS webserver, https://topcons.cbr.su.se/pred/result/rst_0rbd8q9p/ (Tsirigos et al., 2015), predicts no transmembrane domain, but an insertion region of the protein to be lipidated. These predictions corroborate the ones found by LipoP and SMART webservers.

**Figure 1:**
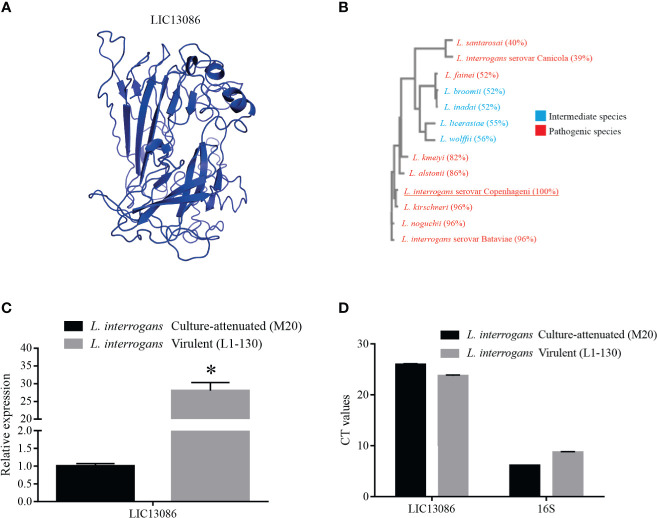
*In silico* analysis of LIC13086 and real-time PCR native expression quantification. **(A)** Tertiary model of LIC13086 generated by molecular modeling in the I-TASSER webserver and visualized by PyMoL. **(B)** Dendogram resulting of multiple alignments performed by Clustal Omega of LIC13086 with other similar sequences of different species of *Leptospira* identified by BLASTp (pathogenic in red; intermediate in blue). **(C)** LIC13086 relative gene expression in culture-attenuated and virulent strains of *L. interrogans* by qPCR. Gene expression was normalized to 16S gene. **(D)** CT values of mRNA expression in culture-attenuated and virulent strains of *L. interrogans*. The bars represent the means of three different experiments in triplicates with the corresponding standard deviation. *Indicates significant difference calculated by Student *t-*test with p-values < 0.05.

The highest-scored 3D model of LIC13086 retrieved by the I-TASSER webserver (**Figure 1A**), based on available protein structures in Protein Data Bank (PDB), presented similarities with a putative calcium-binding lipoprotein of *Bacterioides ovatus* (BACOVA_00967), a fibronectin-binding protein of *Staphylococcus aureus*, and several outer membrane proteins from different microorganisms. When the sequence of LIC13086 was compared with other *Leptospira* species, a multiple sequence alignment resulted in higher identity, equal or higher than 82% with the pathogenic species *L. kirschneri*, *L. interrogans* serovar Batavie, *L. noguchii*, *L. alstonii*, and *L. kmetyi*, while the other pathogenic strains presented percentage similar to the intermediate ones, in the range of 39 to 56% identity (**Figure 1B**). This result indicates higher identity of LIC13086 among pathogenic leptospires.

The evaluation of the comparative gene expression levels of LIC13086 in *L. interrogans* virulent and culture-attenuated strains (L1-130 and M20, respectively), revealed a 28-fold higher relative expression in the virulent leptospires (**Figure 1C**). The 16S gene was used as a control for gene expression normalization (**Figure 1D**).

### Protein Expression and Structural Analysis

**Figure 2:**
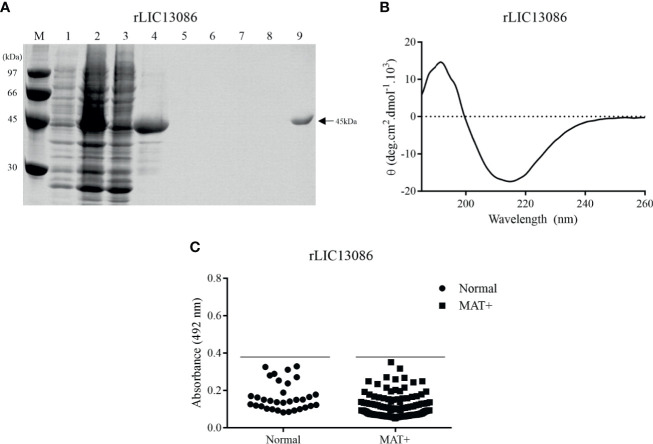
Analysis of rLIC13086 by SDS-PAGE, CD Spectroscopy, and serum reactivity with serum of confirmed leptospirosis. **(A)** Recombinant protein purification samples were separated by SDS-PAGE (12%) and stained with Comassie Blue. M: molecular weight LMW (Ge Healthcare), (1) non-induced total lysates, (2) induced total lysates, (3) induced soluble fraction, (4) induced insoluble fraction, (5–8) washing steps with imidazole (5, 20, 40, and 60 mM), and (9) purified protein after elution. **(B)** CD spectra was recorded as accumulative of 10 scans from 260–190 nm at 25°C and data are expressed in molar residual ellipticity. **(C)** The recombinant protein was immobilized onto ELISA plates (250 ng/well), and the reactivity was assessed by total IgG antibodies in sera from leptospirosis patients at convalescent phase (MAT+) and detection with incubation of HRP-conjugated anti-human IgG (1:5,000). The cutoff values are represented by the horizontal line and defined as the mean plus three standard deviation of the absorbances values from normal human serum (NHS).

The coding sequence of LIC13086 without the N-terminal signal peptide was cloned into pAE expression vector, which adds a 6xHis tag at the C-terminal portion (Ramos et al., 2004). The recombinant protein was expressed in inclusion bodies in *E. coli* BL21 Star™ (DE3) pLysS after IPTG induction. The insoluble protein was denatured in 8 M urea solution and refolded by slow dilution. The refolded protein was purified by immobilized metal affinity chromatography. Recombinant protein and purification steps were analyzed by SDS-PAGE (12%), showing that the recombinant LIC13086 (rLIC13086) exhibited the expected molecular weight of approximately 45 kDa (**Figure 2A**). The structural conformation of rLIC13086 assessed by circular dichroism spectroscopy revealed 11.6% of α-helix, 32.1% of β-sheet, and 56.3% of other structures (**Figure 2B**). No difference of the CD spectrum of the protein was observed in the presence of Ca^2+^ or Mg^2+^, suggesting that there is no interaction of rLIC13086 with these divalent cations (**Figure S1**). Although the 3D model of LIC13086 does not give the percentage of the structures (**Figure 1A**), the CD data is consistent with the predicted α-helix, β-sheet, and other structures, strongly indicating that the protein was refolded from the denatured inclusion bodies.

### Recombinant Protein Reactivity With Confirmed Leptospirosis Serum Samples

The reactivity of rLIC13086 against 150 confirmed leptospirosis serum samples of patients in the convalescent phase (MAT-positive) of the disease was evaluated by ELISA. Considering the cutoff value of 0.38, which was calculated using the normal human sera as the negative control, no reactivity against rLIC13086 was observed in the MAT-positive serum samples (**Figure 2C**). Higher amounts of the LIC13086 protein (250 ng/well versus 1 μg/well) or longer incubation time did not increase the sensitivity of the assay (**Figure S2**). We have used Western blotting to confirm that the cloned LIC13086 protein reacted with mouse anti-LIC13086 with the expected size of the protein (**Figure S3**). Thus, these data show that LIC13086 protein has low immunogenicity against human anti-LIC13086 antibodies. In fact, low immunogenicity was also observed with mice immunization for the generation of polyclonal antiserum, in which very low titer was achieved even after two boosters injections.

### Cellular Location of Protein LIC13086 on *L. interrogans*

**Figure 3:**
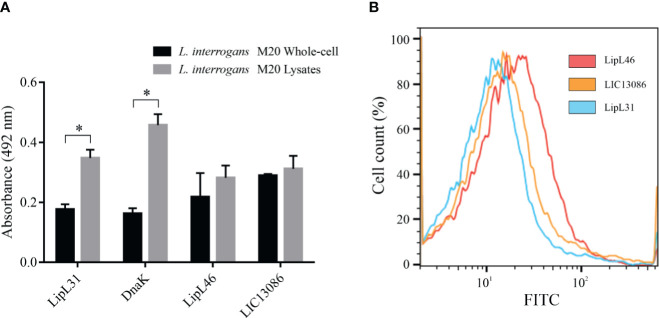
Cellular location of native LIC13086 in *L. interrogans* serovar Copenhageni. **(A)** *L. interrogans* intact cells or soluble lysates were immobilized onto ELISA plates. Primary antiserum against LipL31, DnaK, LipL46, or LIC13086 were added and incubated for 1 h at 37°C. The detection was performed with HRP-conjugated goat anti-mouse IgG (1:5,000). Data represent the means ± the standard deviation of triplicates, representative of three independent experiments. **(B)** *L. interrogans* were fixed with 2% paraformaldehyde at 30°C for 1 h and primary antiserum against LipL31, LipL46, or LIC13086 was added for 1 h at 30°C. After incubation, FITC-conjugated goat anti-mouse IgG (1:50) was added for 16 h at 4°C. Fluorescence measurements were performed in a BD FACSCanto II. *Indicates significant difference calculated by Student *t-*test with p-values < 0.05.

The protein LIC13086 was predicted to be an outer membrane protein by bioinformatics (Yu et al., 2006). Therefore, we performed different assays to verify the cellular location in *Leptospira*. As assessed by ELISA, no significant difference was obtained between the reactivity of immobilized intact cells or lysates against the specific rLIC13086 antiserum (**Figure 3A**), suggesting the outer membrane location. The inner-membrane protein LipL31 and the cytoplasmic DnaK (Haake and Matsunaga, 2002), were used as negative controls. The outer-membrane protein LipL46 (Matsunaga et al., 2006) was used as a positive control. As expected, the absorbances of LipL31 and DnaK increased in the cell lysates compared to the intact cells due to exposure of cytoplasmic proteins, while no significant difference was observed in LipL46, indicating the outer membrane location (**Figure 3A**).

Alternatively, the cellular location was assessed by flow cytometry. Intact leptospires were fixed and incubated with antiserum against LIC13086 or the controls, followed by FITC-conjugated secondary antibodies. LipL31 and LipL46 were used as the negative and positive control, respectively. Cells treated with antiserum against LipL46 or LIC13086 resulted in higher mean fluorescence intensity (MFI) of the leptospiral population when compared to negative control LipL31 (**Figure 3B**), suggesting surface exposure. The calculated MFI was 14.4, 12.8, and 10.1 for LipL46, LIC13086, and LipL31, respectively. Additionally, the fixed leptospires were visualized by confocal microscopy after incubation with antiserum against LIC13086, LipL46, or LipL31 and co-incubation of FITC-conjugated anti-mouse IgG and DAPI. A green fluorescent signal was observed on both the positive control (LipL46) and LIC13086, and the co-localization of FITC-labeled proteins with the DAPI channel was observed (**Figure S4**). In summary, the results of different methods altogether point to the leptospiral outer membrane location of LIC13086.

### Characterization of rLIC13086 Binding to ECM Components

**Figure 4:**
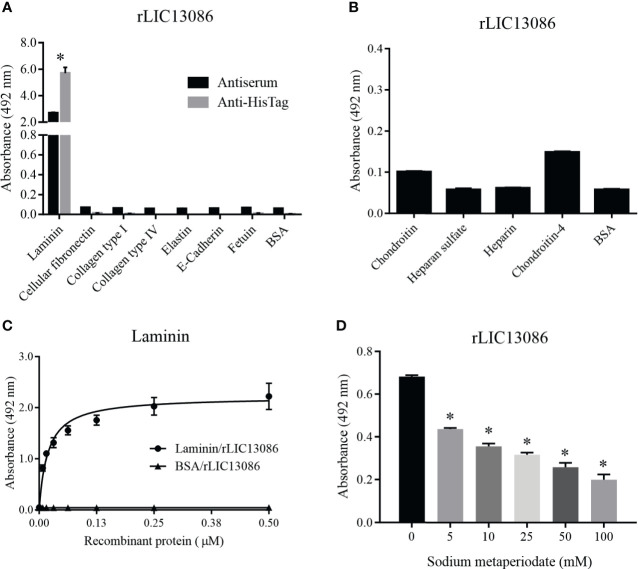
Binding of rLIC13086 with extracellular matrix components. **(A)** Each ECM component was coated onto ELISA plates for 16 h at 4°C and incubated with rLIC13086 (1 μg) for 2 h at 37°C. The detection was performed by incubation with HRP-conjugated anti-HisTag mAb (1:10,000), or anti-rLIC13086 antiserum (1:5,000) and secondary HRP-conjugated anti-mouse IgG (1:5,000). **(B)** Glycosaminoglycans were immobilized onto ELISA plates for 16 h at 4°C, incubated with rLIC13086 (1 μg) for 2 h at 37°C, following detection by HRP-conjugated anti-HisTag mAb (1:10,000). **(C)** Coated laminin (1 μg) was incubated with different concentrations of rLIC13086 (0–0.5 μM) for 2 h at 37°C and detected with HRP-conjugated anti-HisTag mAb (1:10,000). **(D)** Laminin (1 μg) was immobilized onto ELISA plates, incubated with different concentrations of sodium metaperiodate (0–100 mM) and interacted with rLIC13086 (1 μg) at 37°C for 2 h. Detection was performed with HRP-conjugated anti-HisTag mAb (1:10,000). BSA and Fetuin were used as negative controls. The bars represent the means of three different experiments in triplicates with the corresponding standard deviation. * Indicates significant difference calculated by Student *t-*test with p-values < 0.05.

Due to the outer membrane surface location of the protein LIC13086, we evaluated its ability to mediate the direct interaction with the human host ECM proteins. Thereby, laminin, cellular fibronectin, collagen type I, collagen type IV, elastin, e-cadherin, and the controls fetuin and BSA were immobilized onto ELISA plates and rLIC13086 binding was evaluated. The protein rLIC13086 interacted significantly only with laminin when compared with negative controls (**Figure 4A**). In a similar assay, rLIC13086 showed no interaction with various glycosaminoglycans (GAGs), namely chondroitin, heparan sulfate, heparin, and chondroitin-4 (**Figure 4B**). The rLIC13086 binding to laminin was dose-dependent and saturable, with a dissociation constant (K_D_) of 0.0187 ± 0.0023 μM (**Figure 4C**), indicating the specificity of the interaction. Additionally, the oxidation of laminin by sodium metaperiodate significantly reduced the binding of rLIC13086 in a dose-dependent manner (**Figure 4D**), suggesting that the carbohydrate residues are important for the binding of rLIC13086. Altogether, these data indicate that LIC13086 might act as an adhesin mediating *Leptospira*-host interactions.

### Characterization of rLIC13086 Binding to Plasma Components

**Figure 5:**
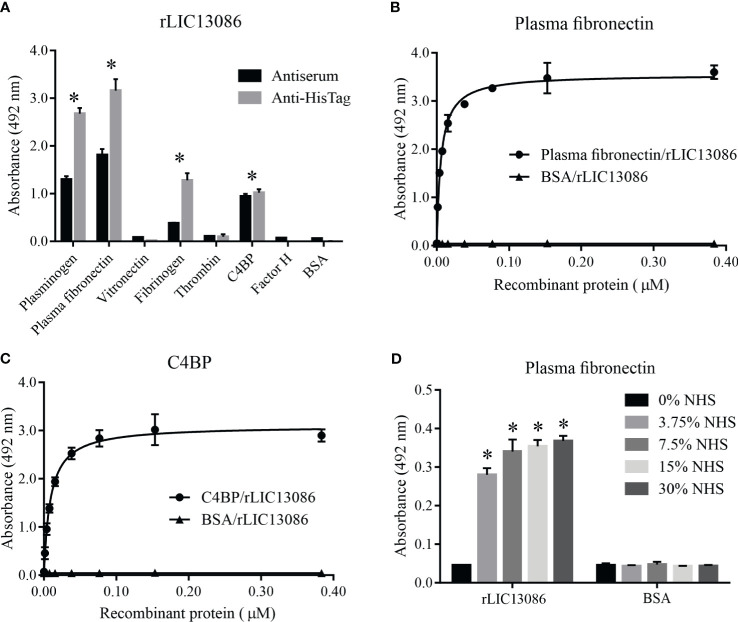
Interaction of rLIC13086 with plasma components. **(A)** Plasma components were coated onto ELISA plates (1 μg) and incubated with rLIC13086 (1 μg) for 2 h at 37°C. Detection was performed with HRP-conjugated anti-HisTag mAb (1:10,000), or anti-rLIC13086 antiserum (1:5,000) and secondary HRP-conjugated anti-mouse IgG (1:5,000). Coated plasma fibronectin **(B)** and C4BP **(C)** were incubated with different concentrations of rLIC13086 (0–0.5 μM) for 2 h at 37°C and detected with HRP-conjugated anti-HisTag mAb (1:10,000). **(D)** rLIC13086 was immobilized and incubated with increasing concentrations of NHS. The interaction was assessed by the addition of rabbit anti-human plasma fibronectin (1:10,000) followed by HRP-conjugated anti-rabbit IgG (1:10,000). BSA was used as negative control. The bars represent the means of three different experiments in triplicates with the corresponding standard deviation. * Indicates significant difference calculated by Student *t-*test with p-values < 0.05.

To evaluate whether LIC13086 can contribute to the wide range of *Leptospira* strategies to escape, invade, and colonize human organs, we tested by ELISA the ability of rLIC13086 to bind to various components of human plasma and complement regulators. The detection was performed both by monoclonal anti-6xHis antibodies or anti-rLIC13086 polyclonal mice antiserum. The recombinant protein was capable of binding plasminogen, plasma fibronectin, fibrinogen, and C4BP when compared with the negative control BSA (**Figure 5A**). Plasma fibronectin and C4BP bindings to rLIC13086 were dose-dependent, with K_D_ of 5.8 ± 0.4 nM and 8.9 ± 0.8 nM, respectively (**Figures 5B, C**). In addition, immobilized rLIC13086 was able to capture plasma fibronectin directly from diluted NHS (**Figure 5D**). Therefore, these interactions might suggest an effect of LIC13086 in the mechanism of *Leptospira* infection establishment.

### Evaluation of rLIC13086 Interaction With Plasminogen

**Figure 6:**
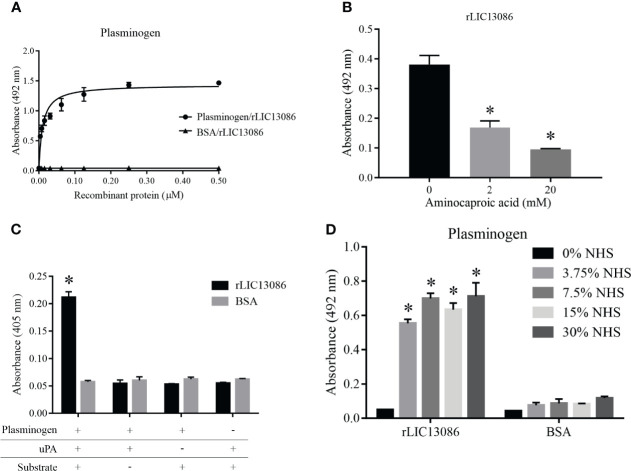
Characterization of rLIC13086 interaction with plasminogen. **(A)** Coated plasminogen (1 μg) was incubated with different concentrations of rLIC13086 (0–0.5 μM) for 2 h at 37°C and detected with HRP-conjugated anti-HisTag mAb (1:10,000). **(B)** The immobilized Plasminogen was co-incubated with aminocaproic acid (0, 2, and 20 mM) and rLIC13086 (1 μg) for 2 h at 37°C. Detection was performed with HRP-conjugated anti-HisTag mAb (1:10,000). **(C)** Coated rLIC13086 was incubated with plasminogen, uPA, and the chromogenic substrate D-val-leu-lys-p-nitroanilide dihydrochloride. Reactions lacking one of each component were used as controls. **(D)** rLIC13086 was immobilized and incubated with increasing concentrations of NHS. The interaction was assessed by the addition of mouse anti-human plasminogen IgG (1:10,000) followed by HRP-conjugated anti-mouse IgG (1:10,000). BSA was used as negative control. The bars represent the means of three different experiments in triplicates with the corresponding standard deviation. *Indicates significant difference calculated by Student *t-*test with p-values < 0.05.

The binding of rLIC13086 to plasminogen was dose-dependent and saturable with the calculated K_D_ of 10.1 ± 1.4 nM (**Figure 6A**). The structural kringle domains of plasminogen are known to mediate the binding with several leptospiral plasminogen-binding proteins *via* the lysine residues (Vieira et al., 2010a; Vieira et al., 2012; Vieira and Nascimento, 2016). In order to investigate the participation of the plasminogen kringle domains in the interaction with rLIC13086, we added the lysine analog aminocaproic acid (ACA) to the reaction as a competitor. Dose-dependent inhibition of rLIC13086 interaction was observed when ACA was added (**Figure 6B**), suggesting that the lysine residues in rLIC13086 probably mediate the interaction to plasminogen.

The zymogen plasminogen is cleaved by physiological specific activators, such as urokinase-type (uPA) or tissue-type (tPA) plasminogen activators, generating the active plasmin. We have previously shown that bacterial plasminogen receptors at the leptospiral surface support the activation of active plasmin in the presence of exogenous physiological activators (Vieira et al., 2010a). To evaluate if plasminogen attached to rLIC13086 can be converted into plasmin, the recombinant protein-bound plasminogen was incubated with uPA and a specific plasmin chromogenic substrate. As observed in **Figure 6C** as a means of substrate cleavage the plasminogen bound to rLIC13086 could be converted into plasmin. The negative controls lacking at least one of the components of the reaction showed no specific enzymatic activity (**Figure 6C**). Moreover, rLIC13086 could capture plasminogen available in NHS *in vitro*, diluted up to 3.75% (**Figure 6D**), suggesting that this mechanism may occur *in vivo* during *Leptospira* infection.

### Evaluation of rLIC13086 Interaction With Fibrinogen

**Figure 7:**
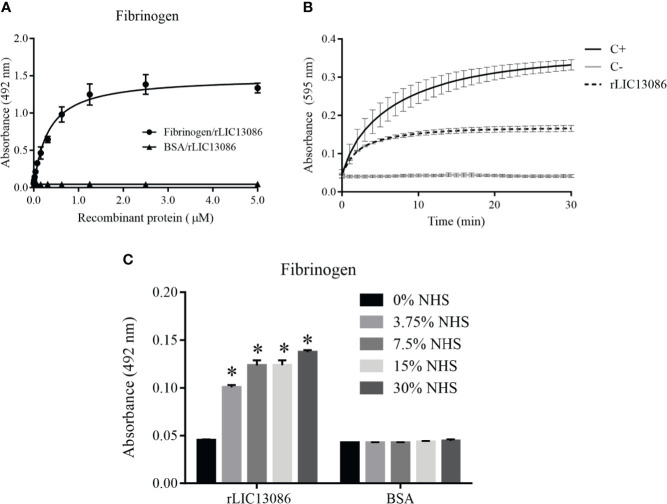
Characterization of rLIC13086 interaction with fibrinogen. **(A)** Coated fibrinogen (1 μg) was incubated with different concentrations of rLIC13086 (0–5 μM) for 2 h at 37°C and detected with HRP-conjugated anti-HisTag mAb (1:10,000). **(B)** rLIC13086 was incubated with human fibrinogen for 2 h at 37°C, thrombin was added, and fibrin clot formation was recorded for 30 min by measuring absorbance at 595 nm. C+: Positive control with only fibrinogen and thrombin; C−: Negative control without fibrinogen. **(C)** rLIC13086 was immobilized and incubated with increasing concentrations of NHS. The interaction was assessed by the addition of goat anti-human fibrinogen (1:10,000) followed by HRP-conjugated anti-goat IgG (1:50,000). BSA was used as negative control. The bars represent the means of three different experiments in triplicates with the corresponding standard deviation. * Indicates significant difference calculated by Student *t-*test with p-values < 0.05.

The rLIC13086 was able to bind fibrinogen and the interaction was dose-dependent and saturable (**Figure 7A**). The calculated dissociation constant K_D_ was 0.3200 ± 0.0318 μM. Endogenous soluble fibrinogen is cleaved by thrombin and converted into insoluble fibrin for clot formation. Therefore we evaluated if rLIC13086 bound to fibrinogen can interfere with the fibrin clot formation process. After interaction with the recombinant protein, fibrinogen was incubated with thrombin and the clot formation was measured over time by absorbance at 595 nm. As a result, rLIC13086 was capable to significantly inhibit the fibrin clot formation when compared with the positive control containing only fibrinogen and thrombin (**Figure 7B**). Furthermore, when incubated with different concentrations of NHS, rLIC13086 could acquire fibrinogen even at low concentrations (3.75% NHS) (**Figure 7C**). The interaction of rLIC13086 with fibrinogen and sera capture *in vitro* might suggest the role of LIC13086 during the pathogenesis and mechanism of fibrin clot inhibition by *Leptospira*.

### Characterization of rLIC13086 Binding to Complement System Components

**Figure 8:**
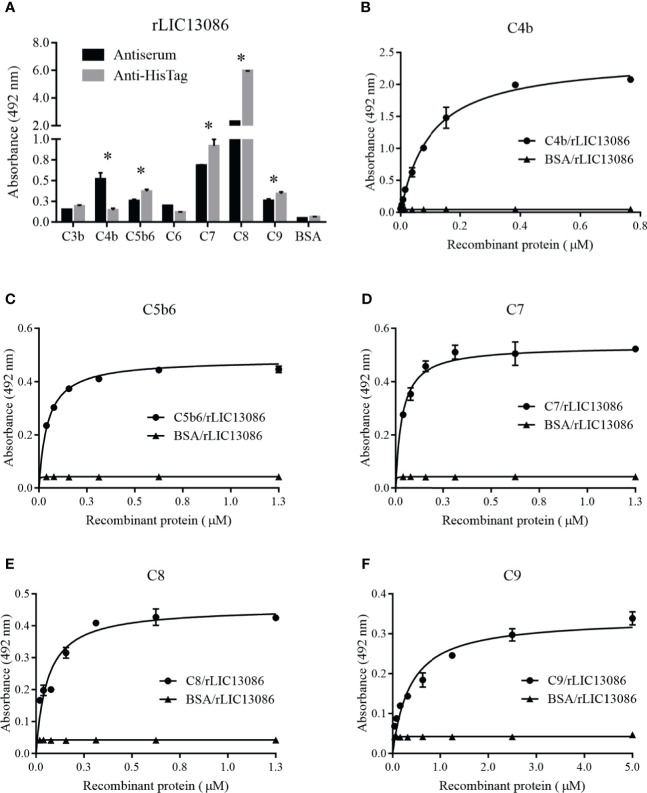
Interaction of rLIC13086 with complement system components. **(A)** Complement system components were coated onto ELISA plates (1 μg) and incubated with rLIC13086 (1 μg) for 2 h at 37°C. Detection was performed with HRP-conjugated anti-HisTag mAb (1:10,000) or anti-rLIC13086 antiserum (1:5,000) and secondary HRP-conjugated anti-mouse IgG (1:5,000). **(B)** Coated C4b, **(C)** C5b6, **(D)** C7, **(E)** C8, and **(F)** C9 were incubated with different concentrations of rLIC13086 (0–5 μM) for 2 h at 37°C and detected with HRP-conjugated anti-HisTag mAb (1:10,000). BSA was used as negative control. The bars represent the means of three different experiments in triplicates with the corresponding standard deviation. * Indicates significant difference calculated by Student *t-*test with p-values < 0.05.

ssPathogenic *Leptospira* can escape the host’s innate immune system and is able to survive and establish infection by interacting or degrading proteins and regulators of the complement system, inhibiting the membrane attack complex (MAC) formation (da Silva et al., 2015; Siqueira et al., 2017). As several proteins of the leptospiral outer membrane have been shown to participate in this mechanism, we examined if rLIC13086 can interact with the complement. The protein rLIC13086 showed significant binding to C4b, C5b6, C7, C8, and C9 when compared with the negative control BSA, confirmed by both anti-HisTag and antiserum detection (**Figure 8A**). The binding interaction was evaluated by increasing the concentration of rLIC13086 and a dose-dependent interaction was observed with C4b (**Figure 8B**), C5b6 (**Figure 8C**), C7 (**Figure 8D**), C8 (**Figure 8E**), and C9 (**Figure 8F**), with the K_D_ of 0.1617 ± 0.0235 μM, 0.0403 ± 0.0119 μM, 0.0358 ± 0.0199 μM, 0.1342 ± 0.0485 μM, and 0.8183 ± 0.231 μM, respectively.

**Figure 9:**
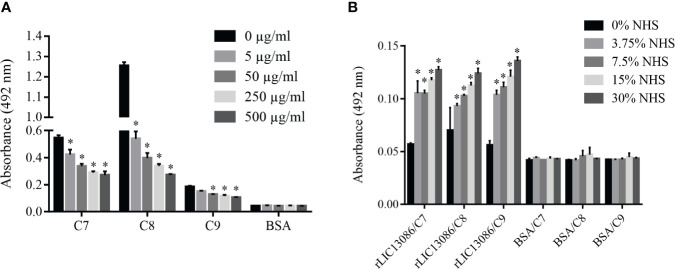
Characterization of rLIC13086 interaction with complement system components. **(A)** rLIC13086 immobilized was co-incubated with C7, C8, or C9 (1 μg) and different concentrations of heparin (0–500 μg/ml) for 2 h at 37°C. Detection was performed with HRP-conjugated anti-HisTag mAb (1:10,000). **(B)** rLIC13086 was immobilized and incubated with increasing concentrations of NHS. The interaction was assessed by the addition of each goat anti-human complement component (1:10,000) followed by HRP-conjugated anti-goat IgG (1:50,000). BSA was used as negative control. The bars represent the means of three different experiments in triplicates with the corresponding standard deviation. * Indicates significant difference calculated by Student *t-*test with p-values < 0.05.

Some of the complement system proteins which interact with rLIC13086 have described heparin-binding domains (Yu et al., 2005). Therefore, we performed a co-incubation assay of rLIC13086 and different concentrations of heparin to assess whether the interaction of these components with recombinant protein occurs *via* heparin sites. Significant inhibition was observed when 5 μg/ml of heparin was added for C7 and C8 binding, while C9 interaction inhibition was observed at 50 μg/ml of heparin (**Figure 9A**), suggesting that binding of rLIC13086 to C7, C8, and C9 may occur *via* heparin-binding sites. In addition, rLIC13086 immobilized onto plates was able to significantly bind C7, C8, and C9 from different concentrations of NHS when compared to the control BSA (**Figure 9B**). These results suggest that binding of rLIC13086 to the complement pathway proteins may be important for leptospiral survival and immune evasion during infection.

**Figure 10:**
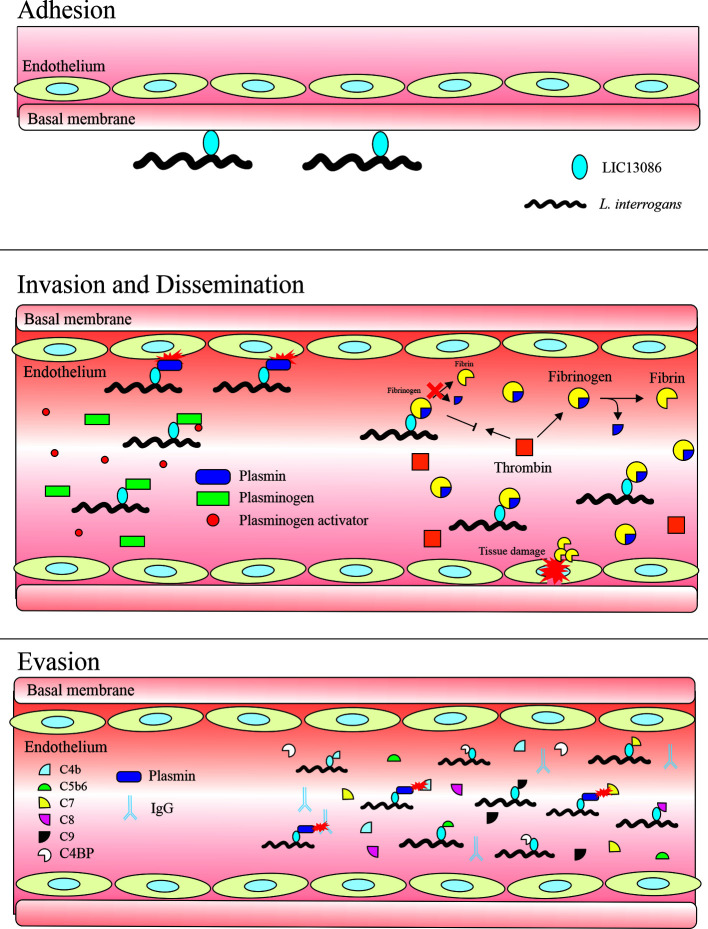
Schematic model representing the possible roles of the protein LIC13086 in *Leptospira*–host interactions and pathogenesis. LIC13086 is a putative lipoprotein experimentally shown to be located in the leptospiral outer membrane. By binding to ECM and basal membrane components, namely laminin, LIC13086 can participate in the adhesion process of leptospires to the host tissues. The binding of LIC13086 to plasminogen/plasmin can contribute to bacterial dissemination, invasion, and immune evasion, through various mechanisms. The plasminogen is first recruited to the bacterial surface *via* bacterial receptors, including LIC13086. The leptospiral-bound plasminogen is converted into plasmin by host-derived plasminogen activators, such as uPA. Plasmin is able to degrade ECM and basal membrane components, resulting in enhanced bacterial penetration and tissue damage. Plasmin also cleaves immunoglobulins and complement molecules, thus favoring immune evasion. By binding to fibrinogen, LIC13086 inhibits its cleavage by thrombin, thus reducing fibrin formation, which can have a consequence in the loss of hemostasis. The complement system comprises various components and regulators. By sequestering the host complement proteins and regulators through various surface proteins, including LIC13086, leptospires can inhibit the complement deposition on the outer membrane, thus avoiding the membrane attack complement (MAC) assembly.

**Figure 10** summarizes the interplay between LIC13086 and the host biological pathways leading to potential pathophysiological consequences.

## Discussion

The analysis of the protein LIC13086 by bioinformatics revealed a domain of unknown function (DUF4842), which is present in the C-terminal portion of a large number of uncharacterized proteins in phylogenetically different organisms such as *Bacteroides*, *Vibrio*, *Shewanella*, and *Leptospira*. The predicted tertiary model of LIC13086 has similarities with *Bacteroides ovatus* protein BACOVA_0067, shows a large β-barrel with an immunoglobulin-like fold, and significant structural similarity to a collagen-bind domain of an *S. aureus* adhesin. Despite the predicted structural similarities between LIC13086 and the calcium-binding protein BACOVA_0067, rLIC13086 did not bind Ca^2+^ when analyzed by circular dichroism.

In whole-genome microarray experiments to evaluate the effects of temperature on gene expression in *L. interrogans* serovar Lai, the LIC13086 homolog (LA3867) exhibited a 9.8 fold-change upregulation when grown at 30°C and shifted overnight to 37°C, when compared to 30°C long-term culture, and a 5.0 fold-change upregulation when compared to 37°C long-term culture (Lo et al., 2006). The temperature shift was performed to evaluate which genes are potentially involved in the early stage of the disease, by mimetizing the temperature change when bacteria enter the host (37°C) from the environment (30°C). Another study, evaluating the *L. interrogans* response to physiologic osmolarity, identified LIC13086 as one of the 25 most strongly upregulated genes when *Leptospira* was grown at physiological osmolarity (Matsunaga et al., 2007). Our results showing the upregulated expression of LIC13086 in virulent strains of *L. interrogans* serovar Copenhageni when compared to culture-attenuated strains, corroborate the previous studies, suggesting that LIC13086 is involved in the colonization of the host and participate in the early stages of leptospirosis pathogenesis. However, rLIC130786 exhibited no reactivity with confirmed leptospirosis serum samples. We hypothesize that these results might be due to the low level of protein expression in *Leptospira*, which was not detected in a quantitative proteomics study by Malmström and colleagues (Malmström et al., 2009), or the low immunogenicity of the protein in humans. Indeed, our results of mice immunization with rLIC13086 for antisera production, corroborate the low immunogenicity of the protein in this animal model, compared to other highly immunogenic proteins, such as LipL32. Low immunogenicity of essential virulence factors can be a mechanism of bacterial immune evasion during an infection.

The gene encoding for LIC13086 is annotated as a LruC domain-containing protein. The LruC protein was identified in *L. interrogans* serovar Pomona as an outer membrane protein conserved amongst pathogenic *Leptospira* and LruC-specific antibody levels were found in higher quantity in eye fluids and sera of uveitic horses when compared with healthy horses, suggesting a role in equine leptospiral uveitis (Verma et al., 2012). The corresponding LruC homolog in *L. interrogans* serovar Copenhageni is LIC20172 (Uniprot ID: Q75FL0), with 100% identity covering 97% of the protein sequence protein in BlastP analysis. Moreover, LIC20172 is described to be a promising vaccine candidate due to the outer membrane location, high conservation, and the presence of predicted B-cells, Helper T-cells, and cytotoxic T-lymphocytes epitopes (Lata et al., 2018). Despite the same annotation and outer membrane location, when aligned by BlastP, LIC13086 and LruC showed only 39.24% identity covering 53% of the sequence, while LIC13086 and LIC20172 showed 38.67% identity covering 89% of the proteins. LruC has only one domain in the N-terminal portion (LruC domain), while LIC20172 has two domains: LruC and the domain of unknown function 4842 (DUF4842). The DUF4842 is similar in sequence between LIC20172 and LIC13086. Therefore, the annotation of LIC13086 as an LruC domain-containing protein is probably misclassified, because LIC13086 does not share similarities with the LruC-domain in LIC20172, sharing similarity only with domain DUF4842.

Several outer membrane proteins of pathogenic *L. interrogans* have been characterized, thus contributing to the understanding of bacterial adhesion, tissue invasion, dissemination, and immune evasion. Some of these proteins exhibit a multifunctional role by binding different components and by acting in different steps of the infection process. Here, we describe a new multifunctional outer membrane protein of *L. interrogans*, LIC13086, which can bind the ECM component laminin, plasma factors, and complement system proteins and regulators.

A well-known mechanism used by leptospires to reach different infection targets and survive during dissemination is the acquisition of host proteases to degrade ECM components and complement proteins. It has been described that pathogenic leptospires can capture circulating plasminogen *via* multiple surface-exposed proteins, which are then converted into plasmin by host activators (Vieira et al., 2010a; Vieira et al., 2012; Kochi et al., 2019; Passalia et al., 2020a). The *Leptospira*-associated plasmin acts by degrading several host molecules during the infection process (Vieira et al., 2013; Vieira and Nascimento, 2016; Vieira et al., 2020b). Our data showing the interaction of rLIC13086 with plasminogen and plasmin suggest the participation of LIC13086 in the pathogen’s dissemination, immune evasion, and penetration during the infection process, although confirmation by *in vivo* experiments employing gene knockout in pathogenic *Leptospira* is needed.

In leptospirosis infection, plasma fibrinogen levels are increased probably associated with severe tissue damage or vascular endothelial injury, and coagulation cascade is highly activated (Chierakul et al., 2008; Wagenaar et al., 2010). Fibrin clot formation is the host response employed to stop bacterial dissemination, however, leptospires can overcome this defense by interacting and sequestering or degrading fibrinogen, thrombin, or other molecules of the coagulation cascade with several outer membrane multifunctional proteins (Oliveira et al., 2013; Fernandes et al., 2015; Passalia et al., 2020b; Vieira et al., 2020). rLIC13086 is a newly characterized multifunctional protein that significantly reduces fibrin clot formation when incubated with fibrinogen *in vitro*. Additionally, binding plasma fibronectin can also interfere with the stabilization of fibrin clot cross-linking. Moreover, the rLIC13086 acquisition of fibrinogen and plasma fibronectin from NHS may reflect what happens *in vivo* and the mechanism by which LIC13086 participates in the reduction of fibrin clot formation. The features describe for rLIC13086 contrast with the previously described leptospiral protein rLIC10774 (Passalia et al., 2020a). The latter is a Ca^2+^-binding protein and although interacting with fibrinogen, does not inhibit fibrin clot formation.

The complement system activation is one of the host immune responses during infection and comprises three main pathways—classical, alternative, and lectin—all resulting in the formation of the MAC (Müller-Eberhard, 1988). The deleterious excessive complement activation must be controlled by different cell surface and soluble regulators (Kim & Song, 2006). It has been demonstrated that pathogenic leptospires have several surface proteins able to bind soluble complement regulatory proteins and terminal complement protein as a mechanism of immune evasion (Meri et al., 2005; Siqueira et al., 2017; Cavenague et al., 2019). Likewise, rLIC13086 exhibited dose-dependent interactions with C4b, C5b6, C7, C8, and C9 molecules of the complement cascade and these bindings may affect the complete MAC formation in the surface of the bacteria. Nevertheless, only C7 and C8 interactions might be significant in physiological conditions. Even so, also by sequestering circulating C4BP, leptospires can use it on the surface as an inhibitor of the complement pathway.

The recombinant protein LIC13086 interacts with ECM laminin with high affinity based on K_D_ value, comparable with the putative adhesins Lsa23, Lsa46, and rLIC10774, previously reported (Domingos et al., 2012; Teixeira et al., 2015; Passalia et al., 2020a). It also interacts with other host plasma factors and exhibits a redundant multiple-binding profile like other characterized outer membrane proteins (Vieira et al., 2014). The property of multiple adhesion partners is observed in other pathogens, such as *Neisseria* spp. and *Yersinia* spp. (Kline et al., 2009; Mikula et al., 2012). The binding affinity to fibrinogen and the resulting effect of rLIC13086 in the inhibition of fibrin clot formation are similar to the reported for Lsa25.6, which showed K_D_ value of 370 nM and resulted in 80% inhibition of fibrin clotting (Pereira et al., 2017). Despite the *in vitro* effect of fibrin inhibition, the K_D_ values of LIC13086 and Lsa25.6 interaction with fibrinogen are relatively high for physiological conditions and *in vivo* experiments with mutants should be considered to confirm this effect. In addition, it has been shown that the proteins rLIC13587 and rLIC13259 are able to reduce the NHS bactericidal effect and inhibit the C9 membrane attack complex formation although interacting with complement components with K_D_ values considered high for physiological conditions (Röttgerding et al., 2017; Cavenague et al., 2019; Kochi et al., 2019). The same effects should be evaluated for LIC13086, since higher binding affinity with complement components was observed.

Difficulties associated in obtaining leptospiral mutants have hampered our understanding of leptospiral pathophysiology (Ko et al., 2009). Only lately, with the construction of shuttle vector *E. coli-Leptospira* and the use of CRISPR-Cas9 methodologies, we started to obtain either knock in and knock out mutants, respectively (Pappas and Picardeau, 2015; Fernandes et al., 2019; Fernandes and Nascimento, 2020; Fernandes et al., 2021). We succeeded in some cases with *L. biflexa* knock in mutants showing the gain of function similar to the ones observed with the *in vitro* studies with the protein LIC11711 (Kochi et al., 2020). Yet, we are encountering many difficulties, probably associated with the features of genes/proteins. This is the case of the present protein, LIC13086, which although cloned in *L. biflexa* and the transcripts detected, no protein expression was observed, impeding our attempt to show gain of function. Attempts to have more mutants are underway.

In any event, our study characterizes a novel multifunctional protein of *L. interrogans*, LIC13086, as an outer membrane protein, highly expressed in virulent strains, with the ability to interact with laminin during the adhesion process, plasma components to facilitate bacterial dissemination and overcome the complement system through binding regulators and complement molecules.

## Data Availability Statement

The original contributions presented in the study are included in the article/**Supplementary Material**. Further inquiries can be directed to the corresponding authors.

## Ethics Statement

The animal study was reviewed and approved by Animal experimentation adopts the guidelines of the Brazilian College of Animal Experimentation (COBEA) and was approved by the Butantan Institute’s Ethics Committee on Animal Use (São Paulo; protocol CEUAIB 3431090117).

## Author Contributions

Conceived and designed the experiments: FP, MV. Performed the experiments: FP, MV. Analyzed the data: MV, FP. Contributed with reagents/materials/analysis tools: MH, MV, AN. Wrote the paper: MV, FP, AN. Revised the paper: all authors. All authors contributed to the article and approved the submitted version.

## Funding

This work was supported by Fundação de Amparo à Pesquisa do Estado de São Paulo (FAPESP), Brazil (grants 2014/50981-0, 2019/17488-2 to AN; 2017/00236-5; 2018/07054-2 to MV; 2017/01102-2 to FP), Conselho Nacional de Desenvolvimento Científico e Tecnológico (CNPq) (grant 301229/2017-1 to AN), São Paulo, Brazil and Fundação Butantan, Sao Paulo, Brazil. The funders had no role in study design, data collection and analysis, decision to publish, or preparation of the manuscript.

## Conflict of Interest

The authors declare that the research was conducted in the absence of any commercial or financial relationships that could be construed as a potential conflict of interest.

## Supplementary Material

Supplementary Figure 1 Recombinant protein reactivity with serum of confirmed leptospirosis. The recombinant protein was immobilized onto ELISA plates **(A)** (250 ng/well) or **(B)** (1,000 ng/well), and the reactivity was assessed by total IgG antibodies (1:100) in sera from leptospirosis patients at convalescent phase (MAT+) and detection with incubation of HRP-conjugated anti-human IgG (1:5,000). The cutoff values are represented by the horizontal line and defined as the mean plus three standard deviations of the absorbances values from normal human serum (NHS).

Supplementary Figure 2 Western-blotting analysis. Recombinant protein rLIC13086 was separated by SDS-PAGE (12%), transferred to a nitrocellulose membrane, and detected by anti-rLIC13086 polyclonal antiserum (1:5,000).

Supplementary Figure 3 Cellular location of LIC13086 by immunofluorescence. *L. interrogans* serovar Copenhageni were fixed with paraformaldehyde (2%), incubated with antiserum against LIC13086 (1:100) produced in mice, and detected with FITC-conjugated goat anti-mouse IgG (1:50). DAPI fluorescent dye was used to stain fixed cells in both samples. Microscope slides were prepared with ProLong Gold Antifade (Invitrogen). Images were captured in confocal immunofluorescence microscope LSM 510 META (Carl Zeiss). C+: Positive control with anti-LipL46, anti-LipL41, and anti-LipL21. C−: Negative control with anti-LipL31.

Supplementary Figure 4 Evaluation of divalent ions in rLIC13086. The protein rLIC13086 was treated with EDTA (3 mM), dialyzed in sodium-phosphate buffer, and supplemented with MgCl_2_ (1 mM) or CaCl_2_ (0.5 or 1 mM) and CD spectra were recorded for each sample. The spectra represent an average of 10 accumulations.
